# Supplementary material for: Beta-Lactam Antibiotic Resistance Genes in the Microbiome of the Public Transport System of Quito, Ecuador
Source: Int J Environ Res Public Health. 2023 Jan 20;20(3):1900. doi: 10.3390/ijerph20031900 (PMC9914694; doi:10.3390/ijerph20031900)

Supplementary Materials

Beta-Lactam Antibiotic Resistance Genes in the Microbiome of the Public Transport System of Quito, Ecuador

Fernanda Hernández-Alomía [1,†], Carlos Bastidas-Caldes [2,3,†], Isabel Ballesteros [1,4], Gabriela N. Tenea [5], Pablo Jarrín-V. [6], C. Alfonso Molina [7,8] and Pablo Castillejo [1,*]

Figure S1. Krona charts depicts bacterial diversity of the most abundant species for Alphaproteobacteria (A), Gammaproteobacteria (B), and Bacilli (C).

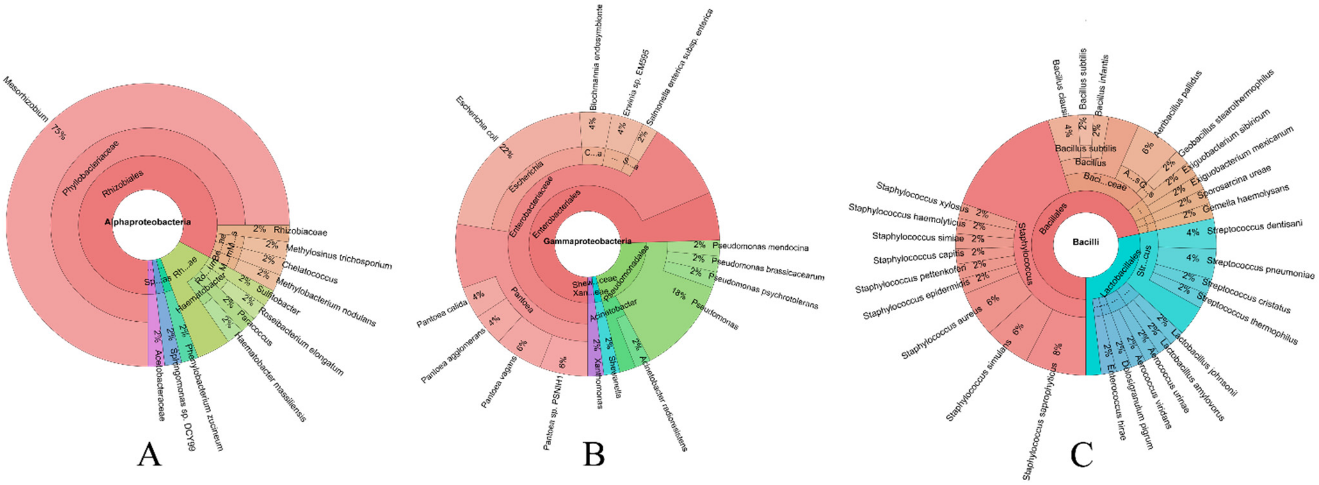

Supplement: Supplementary file 1 [file ijerph-20-01900-s001.zip › Figure S1.pdf]
