# Supplementary material for: Beta-Lactam Antibiotic Resistance Genes in the Microbiome of the Public Transport System of Quito, Ecuador
Source: Int J Environ Res Public Health. 2023 Jan 20;20(3):1900. doi: 10.3390/ijerph20031900 (PMC9914694; doi:10.3390/ijerph20031900)
Supplement: Supplementary file 1 [file ijerph-20-01900-s001.zip › Figure S2.pdf]

Figure S2. Protein variants of TEM-1 (TV1-2), CTX-M1 (CV1-2), and OXA-181 (OV1-2) found in QPT (translated from the nucleotide sequence). Asterisks indicate the residue position of change within the protein.

```
           *116
TV1   PNDERDTTMPAAMATTLRKLL
TV2   PNDERDTTMPVAMATTLRKLL

           *56
CV1   MCSTSKVMAAAAVLKKSESEP
CV2   MCSTSKVMAVAAVLKKSESEP

           *174
OV1   ISATQQIAFLHKLYHNKLHVS
OV2   ISATQQIAFLRKLYHNKLHVS
```
