# Supplementary material for: Beta-Lactam Antibiotic Resistance Genes in the Microbiome of the Public Transport System of Quito, Ecuador
Source: Int J Environ Res Public Health. 2023 Jan 20;20(3):1900. doi: 10.3390/ijerph20031900 (PMC9914694; doi:10.3390/ijerph20031900)
Supplement: Supplementary file 1 [file ijerph-20-01900-s001.zip › Table S1.pdf]

Table S1. *ß*-lactam resistance genes detected from eDNA in QTP stations.

Code	Station	*bla*TEM	*bla*CTXM-1	*bla*OXA-48/181	*mecA*
N1	Terminal La Ofelia	-	-	-	-
N2	La Delicia	+	-	-	-
N3	Del Maestro	+	-	-	+
N4	Vaca de Castro	+	-	-	+
N5	La "Y"	+	-	-	-
N6	San Gabriel	+	-	-	-
N7	Seminario Mayor	+	-	-	-
S1	Terminal Rio Coca	+	-	-	+
S2	Los Sauces	+	-	-	-
S3	Naciones Unidas	+	-	+	-
S4	Eloy Alfaro	+	-	-	+
S5	Baca Ortiz	-	-	-	-
S6	Manuela Cañizares	+	-	-	-
S7	Casa de la Cultura	+	-	-	+
S8	Eugenio Espejo	+	-	-	+
S9	Simon Bolivar	+	+	-	+
S10	Playon La Marin	-	-	-	+
S11	Colegio Montufar	+	-	+	+
S12	Teatro Mexico	+	-	-	+
S13	Terminal El Recreo	+	-	-	-
S14	Ayapamba	+	-	-	+
S15	Terminal Quitumbe	+	-	-	-
S16	Capuli	+	-	-	-
S17	Terminal Sur Guamani	+	-	-	+
C1	Mariana de Jesus	+	-	-	+
C2	Santa Clara	+	-	-	-
C3	La Mariscal	+	+	-	+
C4	El Ejido	+	+	-	+
C5	La Alameda	+	-	-	+
