# Supplementary material for: Beta-Lactam Antibiotic Resistance Genes in the Microbiome of the Public Transport System of Quito, Ecuador
Source: Int J Environ Res Public Health. 2023 Jan 20;20(3):1900. doi: 10.3390/ijerph20031900 (PMC9914694; doi:10.3390/ijerph20031900)
Supplement: Supplementary file 1 [file ijerph-20-01900-s001.zip › Table S2.pdf]

Table S2. Closest match of 16S sequences obtained from QTP and common human microbiota niches after a BLAST search in the GenBank Database

Clone	16S Sequences Accession numbers	Closest match	Identity (%)	Common Human Microbiota Niches	Putative pathogen
1	OP965067	*Staphylococcus epidermidis*	99.79	Skin	Yes
2	OP965068	*Staphylococcus epidermidis*	98.99	Skin	Yes
3	OP965069	*Solirubrobacter* sp.	96.79	None	No
4	OP965070	*Cutibacterium acnes*	100	Skin	Yes
5	OP965071	*Staphylococcus* sp.	100	Skin and mucous membranes	Yes
6	OP965072	*Rubellimicrobium* sp.	96.92	None	No
7	OP965073	*Methylobacterium* sp.	99.04	None	No
8	OP965074	*Staphylococcus epidermidis*	99.37	Skin	Yes
9	OP965075	*Staphylococcus epidermidis*	99.79	Skin	Yes
10	OP965076	*Lysobacter* sp.	99.13	None	No
11	OP965077	Candidatus Saccharibacteria bacterium	97.66	Oral cavity	Yes
12	OP965078	*Streptococcus parasanguinis*	99.59	Respiratory tract	No
13	OP965079	*Exiguobacterium* sp.	99.79	None	No
14	OP965080	*Staphylococcus epidermidis*	98.32	Skin	Yes
15	OP965081	*Staphylococcus epidermidis*	98.57	Skin	Yes
16	OP965082	*Blastococcus* sp.	98.66	None	No
17	OP965083	Acinetobacter lwoffii	99.47	Skin	Yes
18	OP965061	*Pantoea agglomerans*	99.79	None	Yes
19	OP965054	*Pantoea* sp.	99.8	None	Yes
20	OP965066	*Staphylococcus saprophyticus*	100	Gastrointestinal tract	Yes
21	OP965033	*Staphylococcus saprophyticus*	100	Gastrointestinal tract	Yes
22	OP965047	*Staphylococcus simulans*	99.79	Skin	Yes
23	OP965084	*Staphylococcus* sp.	100	Skin and mucous membranes	Yes

24	OP965 115	*Neisseria mucosa*	100	Respiratory tract	Yes
25	OP965 116	*Parvimonas* sp.	99.78	Abscess	Yes
26	OP965 117	*Porphyromonas* sp.	99.16	Oral cavity, gastrointestinal tract, and respiratory tract	Yes
27	OP965 148	*Neisseria mucosa*	100	Respiratory tract	Yes
28	OP965 118	*Neisseria flavescens*	100	Respiratory tract	Yes
29	OP965 131	*Porphyromonas* sp.	99.57	Oral cavity, gastrointestinal tract, and respiratory tract	Yes
30	OP965 119	*Streptococcus downii*	99.38	Oral cavity	No
31	OP965 130	*Lachnoanaerobaculum* sp.	99.77	Oral cavity and gastrointestinal tract	No
32	OP965 120	*Veillonella* sp.	99.79	Oral cavity	Yes
33	OP965 129	*Fusobacterium* sp.	99.54	Oral cavity, respiratory, gastrointestinal, and urinary tracts	Yes
34	OP965 128	*Gemella* sp.	100	Oral cavity and respiratory tract	Yes
35	OP965 127	*Porphyromonas gingivalis*	100	Oral cavity, gastrointestinal tract, respiratory tract, and colon	Yes
36	OP965 126	*Neisseria perflava*	100	Respiratory tract	Yes
37	OP965 125	*Streptococcus* sp.	99.58	Oral cavity and respiratory tract	Yes
38	OP965 124	*Streptococcus* sp.	99.58	Oral cavity and respiratory tract	Yes
39	OP965 123	*Porphyromonas* sp.	100	Oral cavity, gastrointestinal tract, respiratory tract, and colon	Yes
40	OP965 122	*Granulicatella adiacens*	100	Oral cavity	Yes
41	OP965 121	*Streptococcus* sp.	99.79	Oral cavity and respiratory tract	Yes
42	OP965 050	*Pantoea dispersa*	100	None	Yes
43	OP965 027	*Raoultella terrigena*	100	None	Yes
44	OP965 045	*Pantoea agglomerans*	99.57	None	Yes
45	OP965 058	*Pantoea calida*	100	None	Yes
46	OP965 057	*Pantoea calida*	100	None	Yes
47	OP965 161	*Escherichia coli*	100	Gastrointestinal tract	Yes
48	OP965 160	*Escherichia coli*	99.79	Gastrointestinal tract	Yes
49	OP965 164	*Escherichia coli*	99.55	Gastrointestinal tract	Yes
50	OP965 163	*Escherichia coli*	99.78	Gastrointestinal tract	Yes
51	OP965 162	*Escherichia coli*	99.78	Gastrointestinal tract	Yes

52	OP965 062	*Pantoea* sp.	99.79	None	Yes
53	OP965 063	*Pseudomonas psychrotolerans*	100	None	No
54	OP965 026	*Staphylococcus warneri*	99.79	Skin	Yes
55	OP965 042	*Staphylococcus aureus*	100	Respiratory tract	Yes
56	OP965 023	*Aerococcus viridans*	99.19	Skin and respiratory tract	Yes
57	OP965 034	*Staphylococcus warneri*	100	Skin	Yes
58	OP965 085	*Paludibacter propionicigenes*	97.89	None	No
59	OP965 086	*Acinetobacter johnsonii*	99.35	None	No
60	OP965 087	*Dorea longicatena*	99.79	Gastrointestinal tract	No
61	OP965 088	*Lawsonella clevelandensis*	98.65	Abscess	Yes
62	OP965 089	*Veillonella* sp.	98.96	Oral cavity	Yes
63	OP965 090	*Ralstonia pickettii*	99.36	None	Yes
64	OP965 091	*Streptococcus mitis*	99.37	Oral cavity	Yes
65	OP965 092	*Veillonella* sp.	99.8	Oral cavity	Yes
66	OP965 093	*Dorea longicatena*	99.58	Gastrointestinal tract	No
67	OP965 094	*Bacillus* sp.	98.94	None	No
68	OP965 095	*Cutibacterium acnes*	99.78	Skin	Yes
69	OP965 096	*Hymenobacter terrenus*	93.45	None	No
70	OP965 097	*Thermomonas* sp.	99.57	None	No
71	OP965 098	*Corynebacterium* sp.	99.53	Skin and mucous membranes	No
72	OP965 099	*Lawsonella clevelandensis*	99.55	Abscess	Yes
73	OP965 100	*Cutibacterium acnes*	100	Skin	Yes
74	OP965 101	*Corynebacterium freneyi*	100	Skin	No
75	OP965 102	*Streptococcus pseudopneumoniae*	98.95	Respiratory tract	Yes
76	OP965 103	*Janthinobacterium* sp.	99.34	None	No
77	OP965 104	*Propioniciclava soli*	93.19	None	No
78	OP965 112	*Gemmatirosa kalamazoonesis*	91.41	None	No
79	OP965 113	*Exiguobacterium undae*	99.8	None	No

80	OP965 108	*Propioniciclava soli*	93.17	None	No
81	OP965 110	*Veillonella* sp.	99.59	Oral cavity	Yes
82	OP965 111	*Massilia* sp.	98.92	None	No
83	OP965 109	*Nafulsella* sp.	91.67	None	No
84	OP965 107	*Phreatobacter cathodiphilus*	97.38	None	No
85	OP965 106	*Fusobacteriaceae bacterium*	95.1	Oral cavity, respiratory, gastrointestinal, and urinary tracts	Yes
86	OP965 105	Uncultured organism clone	98.28	None	Unkown
87	OP965 114	*Modestobacter* sp.	97.21	None	No
88	OP965 059	*Pseudomonas stutzeri*	100	None	Yes
89	OP965 064	*Lelliottia amnigena*	100	None	Yes
90	OP965 065	*Pantoea* sp.	100	None	Yes
91	OP965 024	*Pantoea eucrina*	100	None	No
92	OP965 043	*Staphylococcus simulans*	100	Skin	Yes
93	OP965 025	*Staphylococcus aureus*	100	Respiratory tract	Yes
94	OP965 044	*Staphylococcus simulans*	99.37	Skin	Yes
95	OP965 035	*Staphylococcus saprophyticus*	100	Gastrointestinal tract	Yes
96	OP965 036	*Bacillus subtilis*	100	None	No
97	OP965 060	*Enterococcus hirae*	99.81	None	Yes
98	OP965 046	*Staphylococcus xylosus*	100	Skin	Yes
99	OP965 159	*Thioclava* sp	100	None	No
100	OP965 158	*Flavobacterium* sp.	97.86	None	No
101	OP965 157	*Lactobacillus johnsonii*	99.6	Gastrointestinal tract	No
102	OP965 156	*Micrococcus luteus*	100	Skin and respiratory tract	Yes
103	OP965 155	*Anaerotignum* sp.	97.67	None	No
104	OP965 154	*Comamonas* sp.	99.64	None	No
105	OP965 153	*Mesorhizobium loti*	99.76	None	No
106	OP965 152	*Rhizobium* sp.	100	None	No
107	OP965 151	*Comamonas aquatica*	99.64	None	No

108	OP965 150	*Shewanella xiamenensis*	99.86	None	Yes
109	OP965 149	*Neisseria mucosa*	100	Respiratory tract	Yes
110	OP965 147	*Pseudomonas plecoglossicida*	99.57	None	Yes
111	OP965 146	*Pseudomonas putida*	99.79	None	Yes
112	OP965 132	*Staphylococcus lugdunensis*	99.79	Skin	Yes
113	OP965 133	*Fusobacterium mortiferum*	99.54	Oral cavity	Yes
114	OP965 134	*Comamonas aquatica*	100	None	No
115	OP965 135	*Micrococcus luteus*	98.97	Skin and respiratory tract	Yes
116	OP965 136	*Rubellimicrobium* sp.	98.27	None	No
117	OP965 137	Uncultured organism clone	99.78	None	Unkown
118	OP965 138	*Methylovirgula* sp.	100	None	No
119	OP965 139	*Comamonas aquatica*	99.78	None	No
120	OP965 140	*Pseudocitrobacter faecalis*	97.41	Gastrointestinal tract and blood	Yes
121	OP965 141	*Haematobacter massiliensis*	100	Respiratory tract	Yes
122	OP965 142	*Pseudomonas plecoglossicida*	98.94	None	Yes
123	OP965 143	*Donghicola* sp.	97.59	None	No
124	OP965 144	*Hymenobacter glacieicola*	99.54	None	No
125	OP965 051	*Pantoea agglomerans*	100	None	Yes
126	OP965 031	*Klebsiella* sp.	100	Skin and gastrointestinal tract	Yes
127	OP965 052	*Enterobacter kobei*	100	Urinary tract	Yes
128	OP965 145	*Pseudomonas* sp.	100	None	Yes
129	OP965 037	*Erwinia gerundensis*	99.57	None	No
130	OP965 030	*Pantoea* sp.	100	None	Yes
131	OP965 053	*Pantoea agglomerans*	99.57	None	Yes
132	OP965 038	*Staphylococcus aureus*	100	Respiratory tract	Yes
133	OP965 039	*Staphylococcus haemolyticus*	100	Skin	Yes
134	OP965 174	*Blastococcus saxobsidens*	98.85	None	No
135	OP965 173	*Cutibacterium acnes*	99.11	Skin	Yes

136	OP965 172	*Cutibacterium acnes*	99.56	Skin	Yes
137	OP965 171	*Cutibacterium acnes*	99.33	Skin	Yes
138	OP965 170	*Paracoccus* sp.	99.52	None	Yes
139	OP965 169	*Corynebacterium tuberculostearicum*	98.87	Skin	Yes
140	OP965 168	*Belnapia* sp.	97.58	None	No
141	OP965 167	*Paracoccus* sp.	98.82	None	Yes
142	OP965 166	*Micrococcus luteus*	99.55	Skin and respiratory tract	Yes
143	OP965 165	*Blastococcus aggregatus*	98.86	None	No
144	OP965 189	*Cutibacterium acnes*	98.9	Skin	Yes
145	OP965 190	*Achromobacter xylosoxidans*	99.36	None	Yes
146	OP965 191	*Tepidomonas* sp.	99.79	None	No
147	OP965 192	*Aeribacillus* sp.	97.55	None	No
148	OP965 193	*Cutibacterium acnes*	99.11	Skin	Yes
149	OP965 194	*Tepidomonas* sp.	99.13	None	No
150	OP965 195	*Cupriavidus metallidurans*	99.14	None	No
151	OP965 196	*Cutibacterium acnes*	99.56	Skin	Yes
152	OP965 197	*Aeribacillus pallidus*	99.17	None	No
153	OP965 198	*Achromobacter xylosoxidans*	99.78	None	Yes
154	OP965 199	*Cupriavidus metallidurans*	99.36	None	No
155	OP965 200	*Cutibacterium acnes*	98.92	Skin	Yes
156	OP965 201	*Chryseobacterium hispanicum*	100	None	No
157	OP965 202	Bacteroidetes bacterium	87.86	None	Yes
158	OP965 203	*Chryseolinea soli*	89.33	None	No
159	OP965 204	*Caldalkalibacillus uzonensis*	96.5	None	No
160	OP965 205	*Escherichia coli*	97.12	Gastrointestinal tract	Yes
161	OP965 206	*Mesorhizobium huakuii*	99.02	None	No
162	OP965 207	*Mesorhizobium huakuii*	99.02	None	No
163	OP965 208	*Mesorhizobium huakuii*	99.01	None	No

164	OP965 209	*Mesorhizobium huakuii*	98.77	None	No
165	OP965 210	*Streptococcus thermophilus*	99.15	None	No
166	OP965 211	*Mesorhizobium terrae*	99.76	None	No
167	OP965 212	*Mesorhizobium huakuii*	100	None	No
168	OP965 213	*Lautropia mirabilis*	99.06	Oral cavity	No
169	OP965 214	*Bacillus* sp.	97.03	None	No
170	OP965 215	*Veillonella* sp.	98.77	Oral cavity	Yes
171	OP965 216	*Mesorhizobium huakuii*	99.02	None	No
172	OP965 217	*Corynebacterium tuberculostearicum*	99.77	Skin	Yes
173	OP965 218	*Brevitalea aridisoli*	86.88	None	No
174	OP965 219	*Nocardioides iriomotensis*	99.29	None	No
175	OP965 220	*Marmoricola* sp.	96.8	None	No
176	OP965 221	*Blastococcus aggregatus*	100	None	No
177	OP965 222	*Corynebacterium simulans*	99.08	Skin	Yes
178	OP965 223	*Lactobacillus crispatus*	99.59	Gastrointestinal tract	No
179	OP965 224	*Aerococcus viridans*	99.57	Skin and respiratory tract	Yes
180	OP965 225	*Cutibacterium acnes*	95.58	Skin	Yes
181	OP965 226	*Citricoccus* sp.	93.56	None	No
182	OP965 227	*Phenylobacterium* sp.	95.26	None	No
183	OP965 228	*Arthrobacter agilis*	99.78	None	No
184	OP965 229	*Novosphingobium silvae*	100	None	No
185	OP965 230	*Mesorhizobium huakuii*	99.04	None	No
186	OP965 231	Clostridiaceae bacterium	94.52	None	No
187	OP965 232	*Escherichia coli*	98.09	Gastrointestinal tract	Yes
188	OP965 233	*Escherichia coli*	98.54	Gastrointestinal tract	Yes
189	OP965 234	*Streptococcus* sp.	100	Oral cavity, and respiratory tract	Yes
190	OP965 235	*Paracoccus* sp.	100	None	Yes
191	OP965 236	*Vagococcus fessus*	98.51	None	Yes

192	OP965 237	*Corynebacterium aurimucosum*	98.14	Urinary tract	Yes
193	OP965 238	*Paracoccus marcusii*	100	None	Yes
194	OP965 239	*Cutibacterium acnes*	99.78	Skin	Yes
195	OP965 240	*Cutibacterium acnes*	99.33	Skin	Yes
196	OP965 241	*Mesorhizobium huakuii*	99.26	None	No
197	OP965 242	*Cutibacterium acnes*	98.88	Skin	Yes
198	OP965 243	*Kocuria palustris*	97.8	None	No
199	OP965 244	*Methyloligella* sp.	93.4	None	No
200	OP965 245	*Pseudomonas* sp.	99.57	None	Yes
201	OP965 246	*Mesorhizobium huakuii*	98.3	None	No
202	OP965 247	*Streptococcus pseudopneumoniae*	100	Respiratory tract	Yes
203	OP965 248	*Mesorhizobium huakuii*	99.51	None	No
204	OP965 249	*Pseudomonas* sp.	99.14	None	Yes
205	OP965 250	*Mesorhizobium huakuii*	98.77	None	No
206	OP965 251	*Mesorhizobium huakuii*	99.03	None	No
207	OP965 252	*Mesorhizobium huakuii*	98.53	None	No
208	OP965 253	*Aeribacillus pallidus*	99.79	None	No
209	OP965 254	*Mesorhizobium huakuii*	99.26	None	No
210	OP965 255	*Mesorhizobium huakuii*	98.07	None	No
211	OP965 256	*Mesorhizobium huakuii*	99.01	None	No
212	OP965 257	*Mesorhizobium huakuii*	97.62	None	No
213	OP965 258	*Mesorhizobium alhagi*	98.598	None	No
214	OP965 259	*Paracoccus* sp.	100	None	Yes
215	OP965 260	*Mesorhizobium alhagi*	97.9	None	No
216	OP965 261	*Kocuria rhizophila*	98.02	None	No
217	OP965 262	*Mesorhizobium huakuii*	98.83	None	No
218	OP965 263	*Mesorhizobium huakuii*	99.02	None	No
219	OP965 264	*Deinococcus budaensis*	94.41	None	No

220	OP965 265	*Kocuria rhizophila*	98.25	None	No
221	OP965 266	*Mesorhizobium terrae*	99.75	None	No
222	OP965 267	*Mesorhizobium loti*	100	None	No
223	OP965 268	*Kocuria rhizophila*	98.46	None	No
224	OP965 269	*Mesorhizobium loti*	100	None	No
225	OP965 270	*Kocuria rhizophila*	98.03	None	No
226	OP965 271	*Kocuria rhizophila*	99.33	None	No
227	OP965 272	*Kocuria rhizophila*	99.77	None	No
228	OP965 273	*Mesorhizobium huakuii*	99.51	None	No
229	OP965 274	*Achromobacter xylosoxidans*	97.93	None	Yes
230	OP965 275	*Kocuria* sp.	99.14	None	Yes
231	OP965 276	*Facklamia* sp.	98.94	None	Yes
232	OP965 277	*Labedella gwakjiensis*	98.42	None	No
233	OP965 278	*Mesorhizobium huakuii*	98.31	None	No
234	OP965 279	*Mesorhizobium terrae*	98.1	None	No
235	OP965 280	*Mesorhizobium huakuii*	98.8	None	No
236	OP965 281	*Mesorhizobium* sp.	99.26	None	No
237	OP965 282	*Cutibacterium acnes*	99.33	Skin	Yes
238	OP965 283	*Mesorhizobium* sp.	99.26	None	No
239	OP965 284	*Mesorhizobium* sp.	99.27	None	No
240	OP965 285	*Mesorhizobium alhagi*	97.88	None	No
241	OP965 286	*Mesorhizobium huakuii*	98.8	None	No
242	OP965 287	*Lawsonella clevelandensis*	98.85	Abscess	Yes
243	OP965 288	*Staphylococcus caprae*	98.3	Blood	Yes
244	OP965 289	*Mesorhizobium huakuii*	99.26	None	No
245	OP965 290	Uncultured organism clone	98.52	None	Unkown
246	OP965 291	*Hymenobacter koreensis*	97.69	None	No
247	OP965 292	*Mesorhizobium terrae*	98.57	None	No

248	OP965 293	*Cutibacterium acnes*	98.04	Skin	Yes
249	OP965 294	*Cutibacterium acnes*	98.26	Skin	Yes
250	OP965 295	*Mesorhizobium huakuii*	99.03	None	No
251	OP965 296	*Luteimonas* sp.	94.32	None	No
252	OP965 183	*Mesorhizobium huakuii*	99.03	None	No
253	OP965 184	*Neobacillus ginsengisoli*	98.09	None	No
254	OP965 185	*Agrococcus* sp.	99.1	None	No
255	OP965 186	*Friedmanniella* sp.	98.38	Gastrointestinal tract	No
256	OP965 187	*Rhodococcus cornyebacterioides*	99.05	None	No
257	OP965 188	*Paracoccus* sp.	98.83	None	Yes
258	OP965 175	*Escherichia coli*	98.93	Gastrointestinal tract	Yes
259	OP965 176	*Escherichia coli*	98.53	Gastrointestinal tract	Yes
260	OP965 177	*Escherichia coli*	98.73	Gastrointestinal tract	Yes
261	OP965 178	*Escherichia* sp.	99.36	Gastrointestinal tract	Yes
262	OP965 179	*Escherichia coli*	99.39	Gastrointestinal tract	Yes
263	OP965 180	*Escherichia coli*	98.54	Gastrointestinal tract	Yes
264	OP965 181	*Escherichia coli*	98.76	Gastrointestinal tract	Yes
265	OP965 182	*Escherichia coli*	98.76	Gastrointestinal tract	Yes
266	OP965 048	*Pantoea agglomerans*	100	None	Yes
267	OP965 049	*Pantoea agglomerans*	100	None	Yes
268	OP965 055	*Pseudomonas* sp.	97.51	None	Yes
269	OP965 056	*Pantoea* sp.	99.57	None	Yes
270	OP965 028	*Leclercia adecarboxylata*	100	None	Yes
271	OP965 029	*Acinetobacter* sp.	99.79	None	No
272	OP965 041	*Staphylococcus warneri*	99.79	Skin	Yes
273	OP965 040	*Staphylococcus saprophyticus*	100	Gastrointestinal tract	Yes
274	OP965 032	*Staphylococcus aureus*	100	Respiratory tract	Yes
275	OP965 301	*Chroococcidiopsis* sp.	97.38	None	No

276	OP965 300	*Vicia faba chloroplast*	99.53	None	No
277	OP965 299	*Vicia faba chloroplast*	99.75	None	No
278	OP965 298	*Colquhounia coccinea* chloroplast	99.76	None	No
279	OP965 297	*Klebsormidium flaccidum* chloroplast	99.76	None	No

Author Contributions: FHA: sample collection, laboratory experiments, data analysis
